# Remdesivir-induced conduction abnormalities: A molecular model-based explanation

**DOI:** 10.3389/jpps.2023.11208

**Published:** 2023-02-13

**Authors:** Ryan Kingsley, Christopher Rohlman, Ashley Otto, Rahul Chaudhary, David Phelan, Robert Kirchoff

**Affiliations:** ^1^ Division of Hospital Medicine, Mayo Clinic, Rochester, MN, United States; ^2^ Department of Chemistry and Biochemistry, Albion College, MI, United States; ^3^ Department of Pharmacy, Mayo Clinic, Rochester, MN, United States; ^4^ Department of Cardiology, UPMC Heart and Vascular Institute, Pittsburgh, PA, United States; ^5^ Division of Infectious Disease, Mayo Clinic, Rochester, MN, United States

**Keywords:** remdesivir, conduction abnormalities, COVID-19, adenosine, GS-441524, cardiac conduction abnormalities

## Abstract

**Purpose:** Remdesivir use in COVID-19 is associated with cardiac conduction abnormalities from unclear mechanisms. A proposed mechanism is the bioaccumulation of the intermediate metabolite GS-441524 resulting in exogenous activation of cardiac adenosine A1 due to the structural similarity between adenosine and GS-441524. The prolonged half-life of GS-441524 can result in sustained activation of adenosine A1 receptors. In this study, we used molecular modeling of adenosine, GS-441524 and the adenosine A1 receptor to assess the potential mechanistic association of the proposed mechanism.

**Methods:** Adenosine and GS-441524 structures were acquired from the PubChem database. Ligand docking was carried out using UCSF Chimera. Models were chosen based on greatest binding affinity and minimum root mean square deviation. Figures of resulting structural models were prepared using UCSF Chimera or PyMOL 2.3.5.

**Results:** By modeling the interaction between the A1 G protein complex and both adenosine and GS-441524, we found that the proposed mechanism of exogenous A1 receptor activation is feasible based on docking compatibility.

**Conclusion:** The proposed mechanism of exogenous cardiac A1 receptor activation from bioaccumulation of GS-441524 as a cause of observed cardiac conduction abnormalities with the use of remdesivir in COVID-19 is viable. Further studies are needed to assess causality.

## Background

**FIGURE 1:**
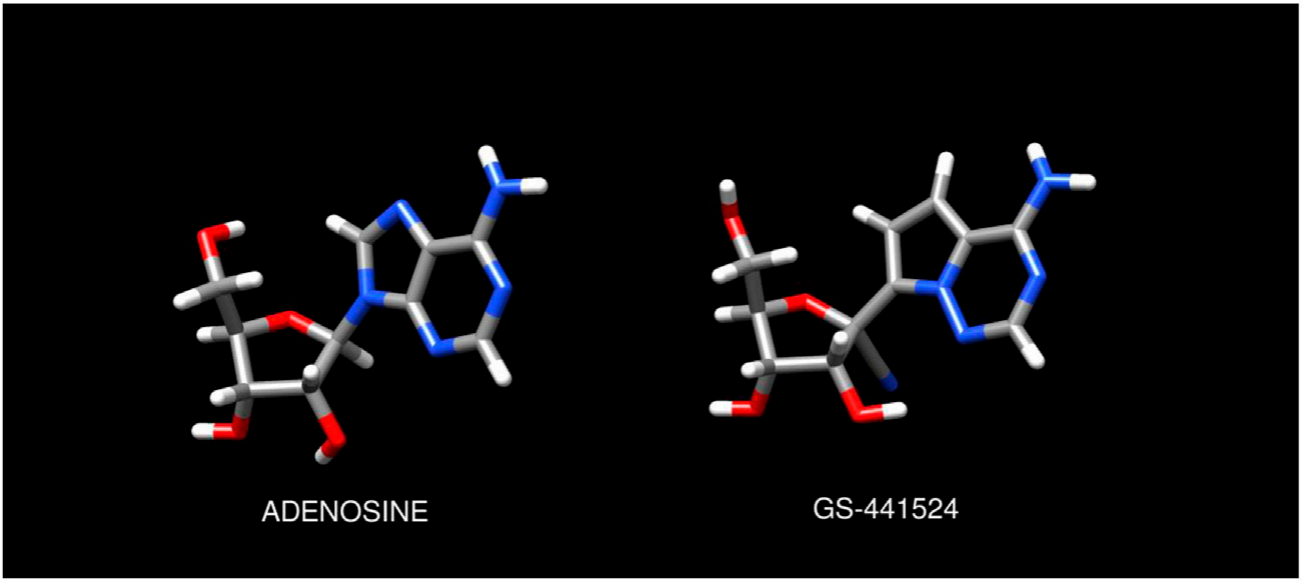
Chemical structures of adenosine and GS-441524 ligands. Molecular models and graphics were created as described in the Methods.

Remdesivir is a nucleoside analog which continues to hold key importance in the management of COVID-19 infection among hospitalized patients (1, 2). Recent literature has reported cardiac conduction abnormalities including PR interval prolongation, QTc prolongation, and profound sinus bradycardia attributed to remdesivir administration (3–7). These effects have been postulated to be a result of exogenous activation of G-protein coupled adenosine A1 receptors by the intermediate metabolite GS-441524(4) due to its prolonged half-life and structural similarity to adenosine as seen in Figure 1.

This paper reviews the relevant pharmacologic characteristics of remdesivir, GS-441524, and adenosine and demonstrates that the proposed mechanism is feasible based on molecular modeling of GS-441524 with the cardiac adenosine receptor.

### Remdesivir pharmacology, pharmacokinetics

Remdesivir, previously known as GS-5734, is a phosphoramidate prodrug of GS-441524, a 1′-cyano-substituted adenine nucleoside analogue. As a phosphoramidate prodrug, intravenous remdesivir is rapidly hydrolyzed in the serum by extracellular kinases to a nucleoside monophosphate (GS-441524), which then undergoes intracellular conversion to the antiviral, pharmacologically active nucleoside triphosphate metabolite (GS-443902). Remdesivir and GS-441524 are bioisosteres of monophosphates, meaning that they can more quickly be activated and undergo intracellular conversion to the active metabolite. By competing with endogenous nucleosides, GS-443902 can incorporate into viral RNA *via* inhibition of RNA-dependent RNA polymerase (8–12).

All remdesivir metabolites confer greater selectivity to RNA-dependent RNA polymerase in comparison to human polymerases (8). The presence of exoribonuclease (ExoN) within virus cells acts to correct RNA chain errors to assist in preventing antiviral activity (13). The activity of remdesivir is only minimally affected by ExoNs, as it can more effectively incorporate into viral RNA than other nucleotide analogs (14, 15). This effective incorporation into RNA allows for improved antiviral activity against single-stranded RNA viruses, including Middle East respiratory syndrome coronavirus (MERS-CoV), severe acute respiratory distress syndrome coronavirus (SARS-CoV) and SARS-CoV-2 (9, 11).

Upon intravenous administration, remdesivir concentrations decline quickly (half-life ∼1 h) as extracellular kinases work to actively metabolize it to GS-441524 (half-life ∼27 h (11, 16). Remdesivir is highly protein bound (80%–90%), though GS-441524 is not (<5%) (16). It acts as a substrate to several cytochrome (CYP) P450 enzymes *in vitro*, including CYP2C8, CYP2D6, and CYP3A4. However, the specifics of these pathways are yet to be quantified but are thought to be minor given the rapid metabolism of the prodrug. GS-441524 is not a substrate of major CYP enzymes, suggesting that GS-441524 does not undergo extensive hepatic metabolism (8, 11, 16, 17). Remdesivir byproducts are predominantly excreted *via* urine, primarily as GS-441524 (49%), followed by remdesivir (10%) and GS-704277 (2.9%). Excretion *via* feces is negligible (16).

### Pharmacologic activity of adenosine on A1 receptors

Adenosine is an endogenous purine nucleoside. It is composed of an adenine molecule with a ribose sugar moiety and is an essential component of adenosine triphosphate (ATP) and cyclic adenosine monophosphate (cAMP) (18, 19). Adenosine activity in the body is widespread, including activity to reduce blood pressure and heart rate, regulation of the sympathetic nervous system, and induction of vasodilation (18).

Adenosine binds to four receptor subtypes that are found on the surfaces of most cells within the body: A1, A2A, A2B, and A3. A1 receptor activation leads to negative chronotropic and dromotropic effect, A2 receptor activation leads to inotropic effects and vasodilation, and A3 receptors are minimally expressed in the myocardium. The adenosine A1 receptors are G_i_ protein-coupled receptors on cell surfaces with inhibitory functions when activated, leading to negative chronotropic and dromotropic effects (18, 20, 21). A1 receptors are expressed in the brain, spinal cord, kidney, spleen, and heart and have a strong affinity for adenosine (20).

In the heart, adenosine binds to A1 receptors primarily expressed in the atria, leading to decreased cAMP production, inhibition of protein kinase A and voltage-gated calcium channels, and subsequent opening of ATP-sensitive potassium gated channels. The inhibition of this calcium influx and increased potassium current mediates the inhibition of atrioventricular (AV) node conduction, causing a shortened action potential duration with refractoriness (18, 20, 21).

## Methods

### Ligand binding docking and structural modeling

The human adenosine A1 receptor-Gi2-protein complex (PDB ID: 6D9H) (22) was used as the target receptor. The 3D structural coordinates for both adenosine (compound CID 60961) and GS-441524,(2R,3R,4S, 5R)-2-(4-aminopyrrolo[2,1-f][1,2,4]triazin-7-yl)-3,4-dihydroxy-5-(hydroxymethyl)oxolane-2-carbonitrile (Compound CID 44468216) structures were acquired from the PubChem database at the NIH NCBI (23). GS-441524 was the only metabolite tested due to the lack of structural plausibility for the other metabolites. Prior to modeling, both ligand structures were minimized using WebMO (24). Ligand docking was carried out using UCSF Chimera (25) and AutoDock Vina (26). The AutoDock Vina search volume was centered on the A1 adenosine binding site, as described previously (22). The search volume dimensions were set to encompass both the putative binding site and the adjacent trans-membrane helical protein regions. Maximum binding modes was set at ten, with a maximum energy difference of 2 kcal/mol. Models were chosen on the basis of greatest binding affinity and minimum root mean square deviation. Figures of resulting structural models were prepared using UCSF Chimera or PyMOL 2.3.5. Datasets are available upon request: The raw data supporting the conclusion of this article will be made available by the authors, without undue reservation.

### Ligand binding docking and structural modeling—Results

**FIGURE 2:**
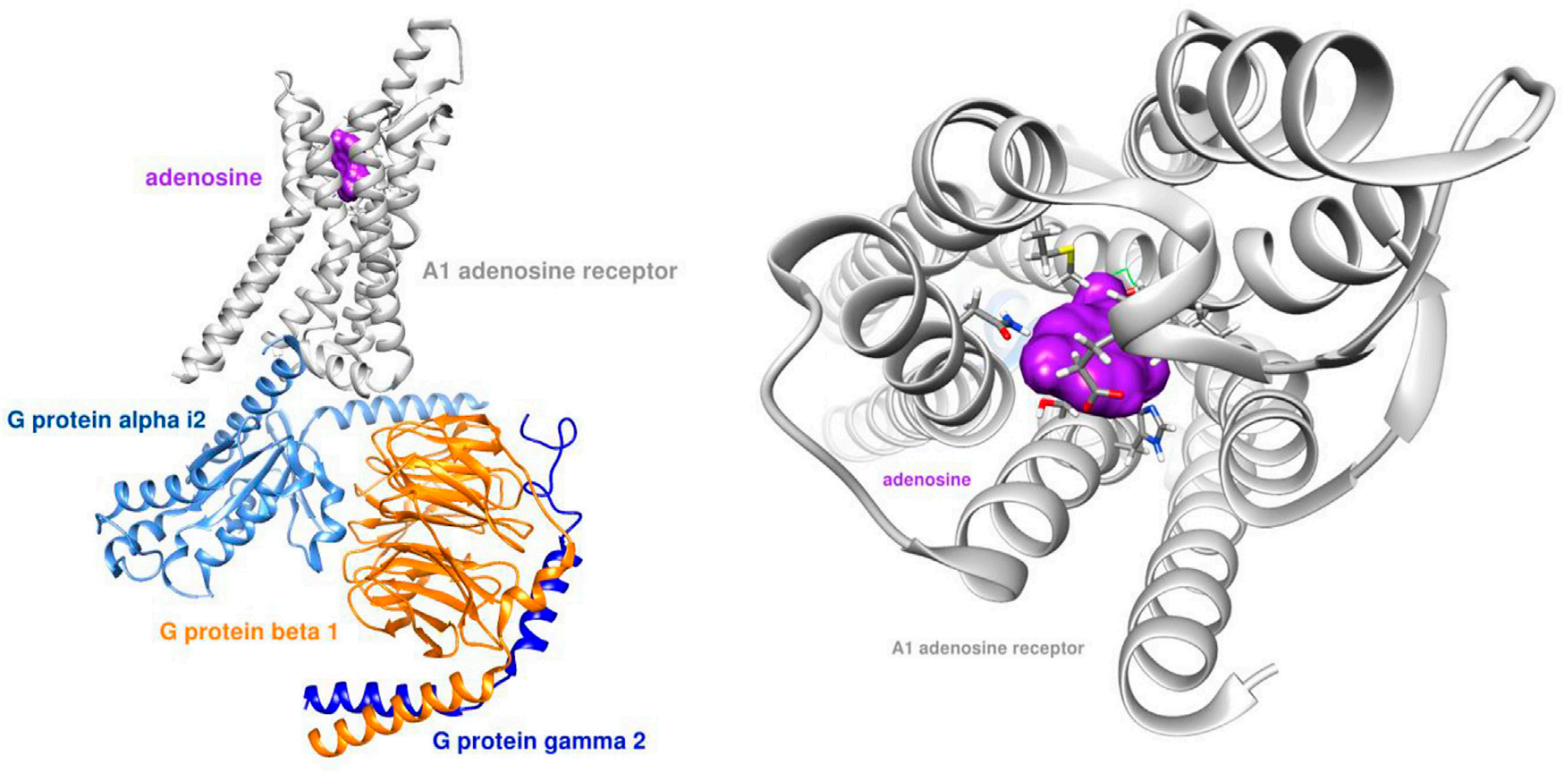
Adenosine receptor - G protein complex (PDB ID: 6D9H) with adenosine ligand.

Our team simulated the interaction between both adenosine and GS-441524 with the A1 receptor. Figure 2 shows the adenosine molecule (purple) binding within the G protein complex.

**FIGURE 3:**
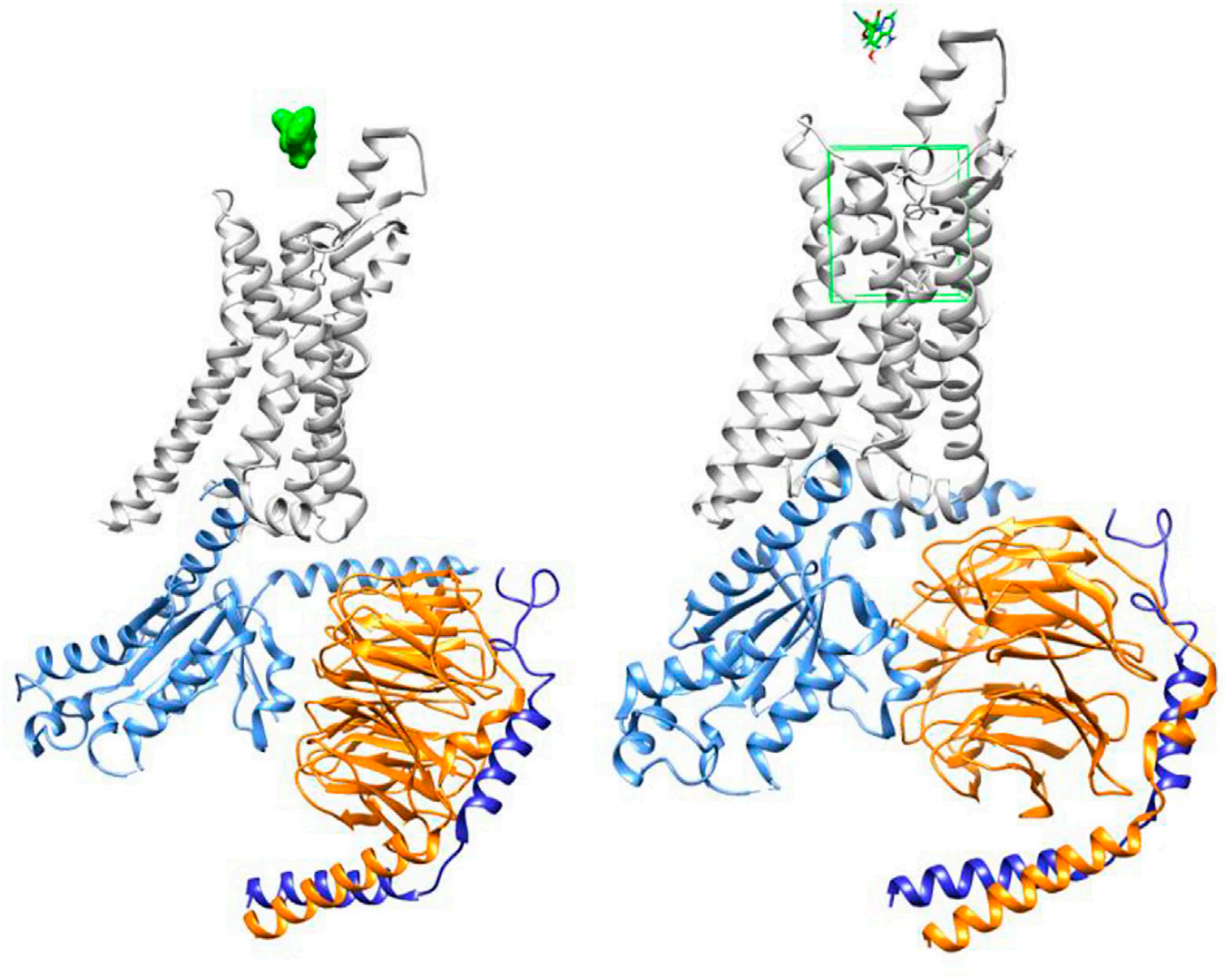
Adenosine receptor - G protein complex (PDB ID: 6D9H) with GS-441524 ligand/Pre-docking.

Figure 3 demonstrates GS-441524 (green) in its pre-docking state with the A1 G protein complex. The adenosine docking site is highlighted in this figure by a green square.

**FIGURE 4:**
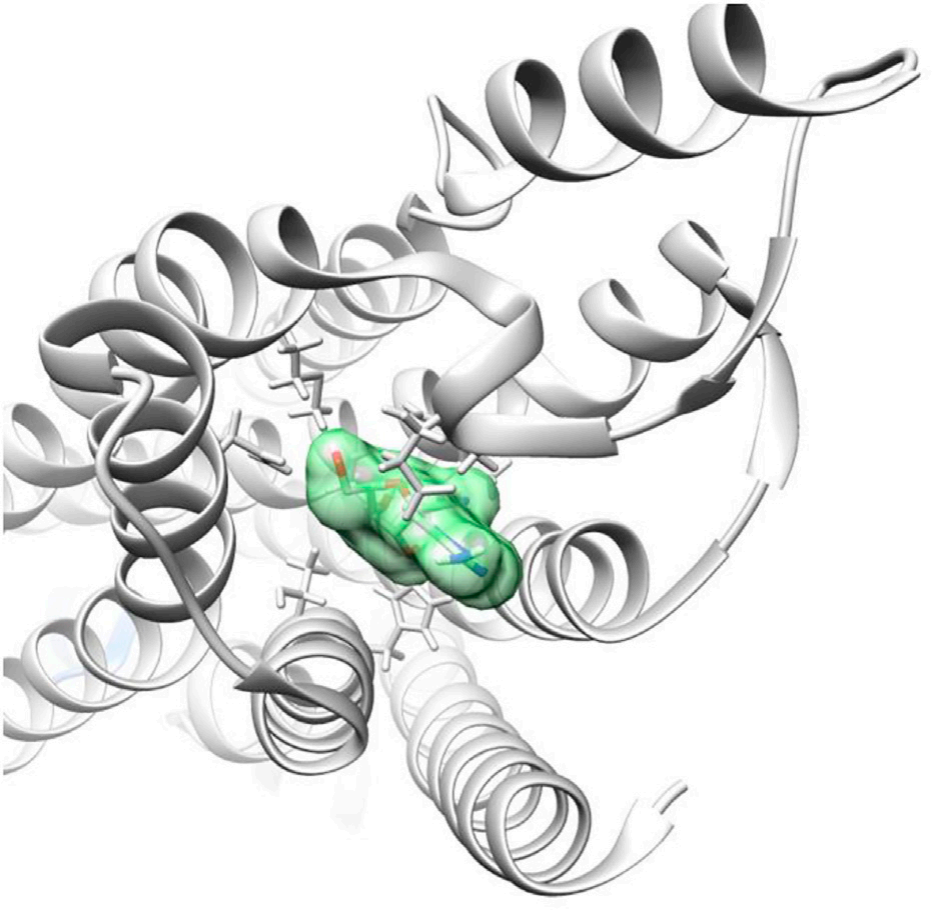
Adenosine receptor - G protein complex with GS-441524 ligand/Post-docking – Axial view.

**FIGURE 5:**
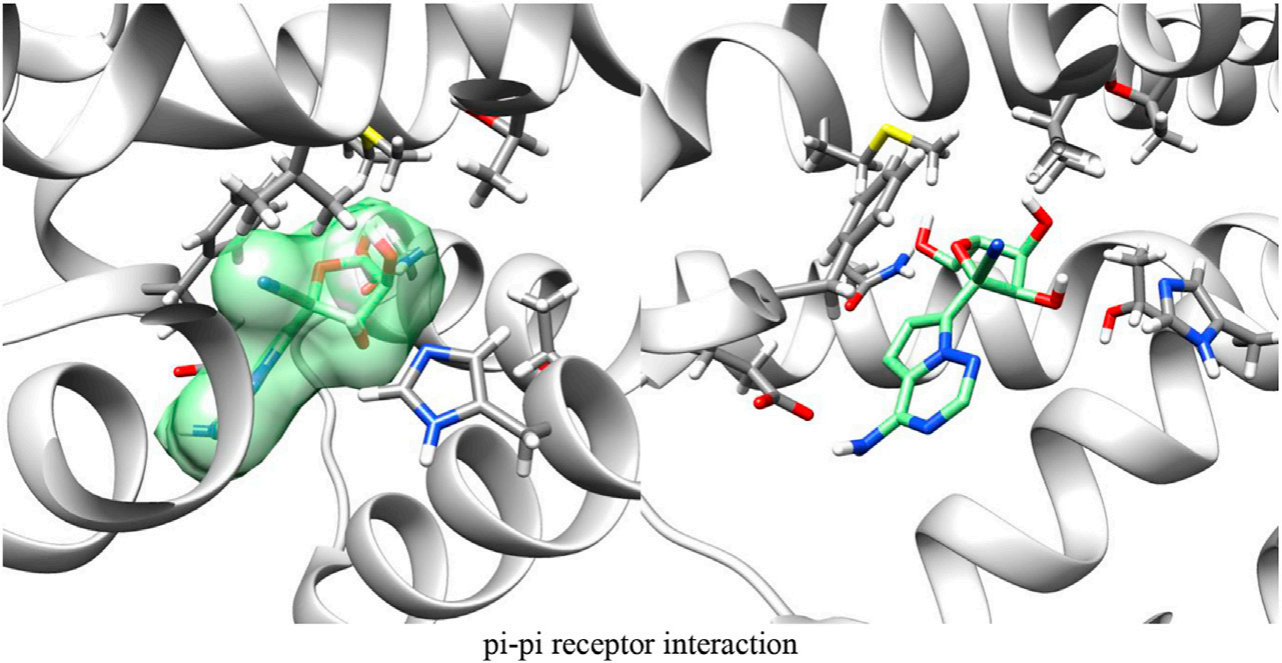
Adenosine receptor - G protein complex with GS-441524 ligand post-docking.

Figures 4, 5 demonstrate GS-441524 (green) in its docking state within the A1 G protein complex.

By modeling the interaction between the A1 G protein complex and both adenosine and GS-441524, we found that the proposed mechanism of exogenous A1 receptor activation is feasible based on docking compatibility.

## Discussion

Molecular modeling described above demonstrates a high level of binding affinity for GS-441524 as an exogenous ligand of the adenosine A1 receptor. Bioaccumulation of GS-441524 is postulated to result in transient, exogenous activation of cardiac adenosine A1 receptors. Stimulation of these receptors is known to have a myocardial depressant effect by slowing conduction and suppressing cardiac pacemaker function (21). This hypothesis is supported by clinical evidence of negative chronotropy, AV conduction delay, ventricular depolarization delay. In our study, we provide supporting evidence for bioaccumulation of GS-441524 as an important factor in the observed incidence of cardiac conduction abnormalities and proarrhythmic effects in patients treated with remdesivir for COVID-19.

There are several potentially important contributors to cardiac conduction delay in these cases and is likely multi factorial. Conduction abnormalities appear to be more common in those with baseline cardiac conduction system disease (e.g., left bundle branch block, right bundle branch block, etc.) (27). Additionally, both hypoxia and inflammation have been shown to induce increased adenosine metabolism and signaling (28, 29). We hypothesize that the well-characterized cytokine-mediated inflammatory state and the hypoxia induced by COVID-19 increases the endogenous adenosine, which in combination with exogenous GS-441524 bioaccumulation results in transient conduction delay. Our modelling approach provides novel insights into the effects of remdesivir and increases the likelihood of a potential mechanistic explanation of cardiac conduction abnormalities observed with COVID-19 and remdesivir use. Further studies are needed to confirm our findings.

## Data Availability

The original contributions presented in the study are included in the article/supplementary material, further inquiries can be directed to the corresponding author.
